# Fabrication of Si heterojunction solar cells using P-doped Si nanocrystals embedded in SiN_*x*_ films as emitters

**DOI:** 10.1186/1556-276X-8-457

**Published:** 2013-11-05

**Authors:** Ping-Jung Wu, Yu-Cian Wang, I-Chen Chen

**Affiliations:** 1 Institute of Materials Science and Engineering, National Central University, Jhongli 320, Taiwan

**Keywords:** Si nanocrystals, Heterojunction solar cells, Emitter

## Abstract

Si heterojunction solar cells were fabricated on p-type single-crystal Si (sc-Si) substrates using phosphorus-doped Si nanocrystals (Si-NCs) embedded in SiN_*x*_ (Si-NCs/SiN_*x*_) films as emitters. The Si-NCs were formed by post-annealing of silicon-rich silicon nitride films deposited by electron cyclotron resonance chemical vapor deposition. We investigate the influence of the N/Si ratio in the Si-NCs/SiN_*x*_ films on their electrical and optical properties, as well as the photovoltaic properties of the fabricated heterojunction devices. Increasing the nitrogen content enhances the optical gap E_04_ while deteriorating the electrical conductivity of the Si-NCs/SiN_*x*_ film, leading to an increased short-circuit current density and a decreased fill factor of the heterojunction device. These trends could be interpreted by a bi-phase model which describes the Si-NCs/SiN_*x*_ film as a mixture of a high-transparency SiN_*x*_ phase and a low-resistivity Si-NC phase. A preliminary efficiency of 8.6% is achieved for the Si-NCs/sc-Si heterojunction solar cell.

## Background

Materials consisting of silicon nanocrystals (Si-NCs) embedded in a dielectric matrix are one promising candidate to realize Si-based third-generation photovoltaic devices owing to their potential benefits of utilizing the visible light of terrestrial solar spectrum and overcoming the efficiency limit of crystalline Si (c-Si) solar cells [1-5]. Sub-stoichiometric Si-based dielectric materials, such as SiO_*x*_, SiN_*x*_, and SiC_*x*_, have been investigated for synthesis of Si-NCs [6-11]. The formation of Si-NCs is based on phase segregation and crystallization in Si-rich dielectric films during the post-annealing process [12].

The low conductivity of Si-NCs embedded in dielectric films limits their applications for the manufacturing of optoelectronic devices. For this reason, impurity doping in Si-NCs embedded in SiO_2_ has been demonstrated to modify the electrical properties of the layers, although there is some debate about the feasibility of doping in Si-NCs [13,14]. In addition to impurity doping, the choice of the surrounding dielectric matrix also plays a crucial role in charge carrier transport. Although the formation of Si-NCs in the SiO_2_ matrix has been investigated in detail [12,15], the carrier transport ability in the Si-NC network is generally insufficient due to the large energy barrier of the surrounding oxide matrix. Charge carrier transport through narrower bandgap dielectrics, such as Si_3_N_4_ or SiC, seems to be more feasible. Compared with SiO_2_ and SiC, Si_3_N_4_ may offer a compromise as a dielectric matrix for the Si-NC network used in solar cell applications since it possesses a medium bandgap (approximately 5.3 eV) which could reduce the energy barrier for carrier transport and also effectively avoid parasitic absorption. However, doped Si-NCs embedded in a SiN_*x*_ matrix (Si-NCs/SiN_*x*_) have not received much attention.

In this work, we present initial fabrication and characterization results of Si heterojunction solar cells using P-doped Si-NCs/SiN_*x*_ films as emitters. The P-doped Si-NCs/SiN_*x*_ films were prepared by electron cyclotron resonance chemical vapor deposition (ECRCVD) followed by high-temperature annealing, and the influence of the chemical composition (N/Si ratio) on their physical properties was investigated. The photovoltaic properties of the fabricated heterojunction devices were also examined as a function of the N/Si composition ratio in the P-doped Si-NCs/SiN_*x*_ films.

## Methods

Fifty-nanometer-thick, homogeneous Si-rich silicon nitride (SRN) films containing phosphorus were deposited by a homemade ECRCVD system on single-side polished p-type (100) single crystalline Si (sc-Si) substrates with a thickness of 675 μm and a resistivity in the range of 5 to 20 Ω cm. Before placing into the deposition chamber, Si substrates were cleaned with acetone and rinsed in deionized water followed by removal of native oxide on Si wafers using a diluted HF dip (5%). The mixed SiH_4_, N_2_, Ar, and PH_3_ gases were then introduced into the deposition chamber at 10 mTorr for film growth. The applied microwave power and the substrate temperature were kept on 700 W and 200°C, respectively. In order to study the influence of the Si/N ratio on film properties, both SiH_4_ and PH_3_ flow rates were kept constant during film growth, while the gas mix ratio (*R*_c_) defined as N_2_/SiH_4_ was varied in the range 0.73 ≤ *R*_c_ ≤ 0.83. The formation of Si-NCs in as-deposited SRN films was carried out by post-growth annealing in a quartz tube furnace at 950°C in N_2_ ambient. Samples with a 1 cm × 1 cm area were used for subsequent fabrication of heterojunction solar cells. Aluminum films deposited by electron gun evaporation were used as contact electrode layers in the solar cells. The front contact on top of the Si-NCs/SiN_*x*_ film was defined by a shadow mask during Al deposition, while the rear contact covered the full back area of the cell. After metallization, the samples were heated at 500°C for 3 min to improve the electrical properties of the contacts.

For the characterization, the bonding configurations of the Si-NCs/SiN_*x*_ films were identified by X-ray photoelectron spectroscopy (XPS). Micro-Raman spectroscopy and transmission electron microscopy (TEM) were used to investigate the crystallization behavior in SRN films after post-growth annealing. Fused quartz wafers were used as substrates for Raman studies to avoid the signal contribution from Si substrates during Raman measurements. X-ray diffraction (XRD) was used to evaluate the Si-NC size of annealed samples. The photovoltaic properties of the fabricated heterojunction solar cells were evaluated at room temperature based on the illuminated current density versus voltage (*J*-*V*) and the internal quantum efficiency (IQE) characteristics under 1-sun air mass 1.5 global spectrum.

## Results and discussion

**Figure 1:**
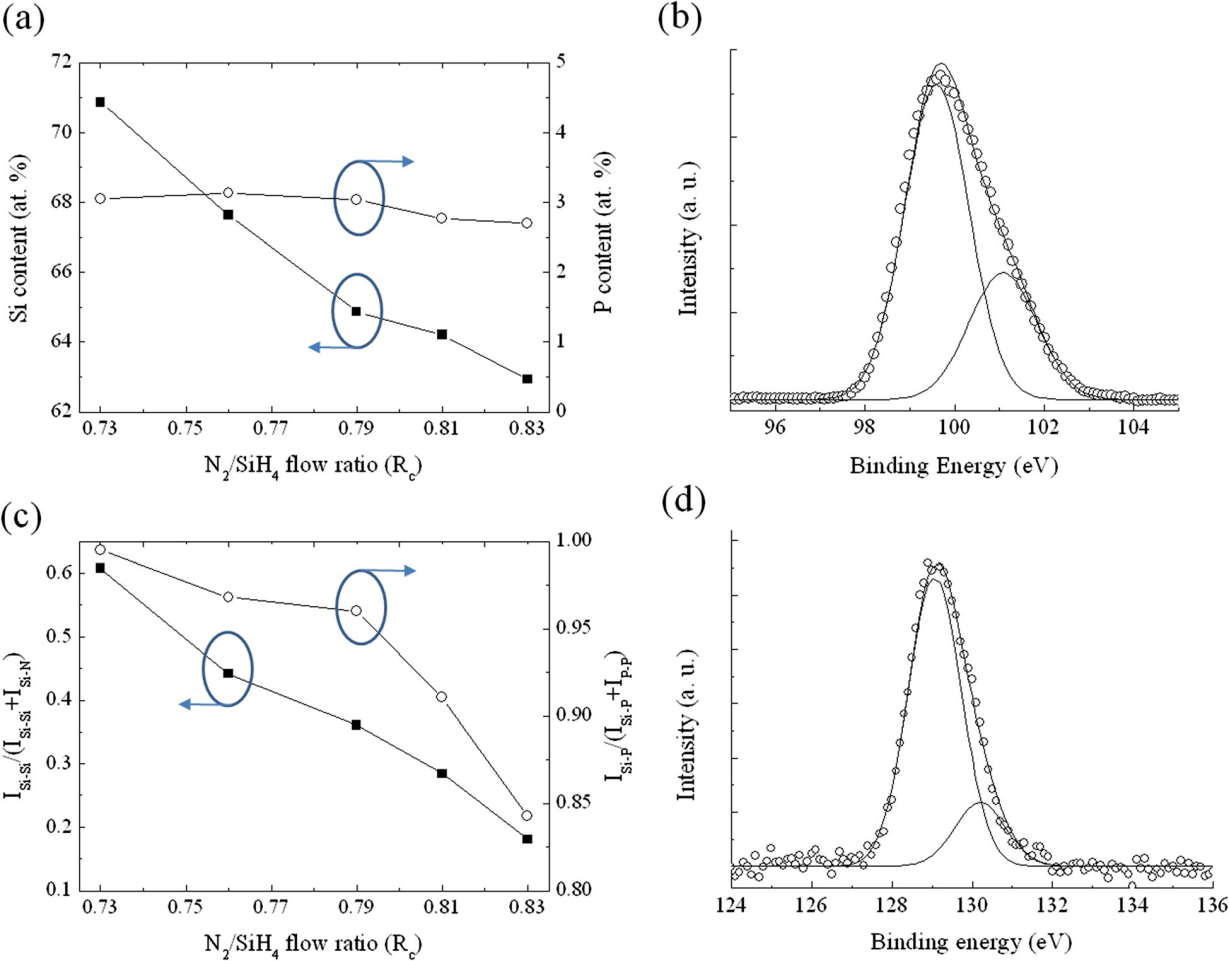
**XPS analysis of P-doped Si-NCs/SiN**_***x***_**films. (a)** Si and P concentrations in P-doped Si-NCs/SiN_*x*_ films as a function of the *R*_c_ value. **(b)** Deconvolution analysis of a representative Si 2*p* XPS spectrum of the P-doped Si-NCs/SiN_*x*_ sample with *R*_c_ = 0.79. **(c)** XPS peak intensity ratios of *I*_Si-Si_/(*I*_Si-Si_ + *I*_Si-N_) and *I*_P-P_/(*I*_Si-P_ + *I*_P-P_) of P-doped Si-NCs/SiN_*x*_ films as a function of the *R*_c_ value. **(d)** Deconvolution analysis of a representative P 2*p* XPS spectrum of the P-doped Si-NCs/SiN_*x*_ sample with *R*_c_ = 0.79.

The relative elemental composition of the P-doped Si-NCs/SiN_*x*_ films was estimated from XPS spectra. The calculation of the chemical composition is based on the integrated area under the N 1 *s*, Si 2*p*, and P 2*p* peaks in conjunction with the sensitivity factors for the elements [16]. Figure 1a shows Si and P concentrations in the samples as a function of the *R*_c_ value. The Si concentration decreases from 70.8 to 62.9 atomic percent (at.%) with the N_2_/SiH_4_ flow ratio adjusted from 0.73 to 0.83, while the P concentration is kept around 3 at.% since the PH_3_/SiH_4_ flow ratio was kept constant during film growth. In order to obtain efficient carrier extraction, a photovoltaic device generally requires the presence of a p-n junction for carrier separation. Thus, active doping of phosphorus in Si-NCs is required for Si-NCs/sc-Si heterojunction solar cells. In this study, XPS was also used to study the chemical structure of P-doped SRN films after post-growth annealing. Figure 1b shows the Si 2*p* XPS spectrum of a representative SRN sample with *R*_c_ = 0.79 after annealing. The deconvolution of the Si 2*p* signal consists of two peaks centered around 99.6 and 101.3 eV, which correspond to elemental Si and Si coordinated in the SiN_*x*_ network, respectively [17]. The analysis of the Si 2*p* peak indicates that the excess Si atoms precipitate out from the dielectric network, leading to the phase separation and formation of Si-NCs. The change in the XPS peak intensity ratio *I*_Si-Si_/(*I*_Si-Si_ + *I*_Si-N_) was applied to investigate the influence of the N/Si ratio on the phase separation in annealed SRN films. As expected, the *I*_Si-Si_/(*I*_Si-Si_ + *I*_Si-N_) decreases with increasing *R*_c_ value (shown in Figure 1c), implying that both phase separation and Si crystallization are reduced in the sample with a lower excess Si concentration. The P 2*p* XPS signal of the annealed SRN film could be deconvoluted into two peaks centered around 129.2 and 130.3 eV (shown in Figure 1d), which are assigned to P atoms surrounded in part with Si atoms and pure phosphorous, respectively [17]. As depicted in Figure 1c, the value of *I*_Si-P_/(*I*_Si-P_ + *I*_P-P_) decreases when increasing the N_2_/SiH_4_ flow ratio. It is suggested that the concentration of the Si-P bond is proportional to the excess Si concentration, implying that phosphorus atoms may exist inside the Si-NCs or at the interfaces between Si-NCs and the SiN_*x*_ matrix in the form of Si-P bonds.

**Figure 2:**
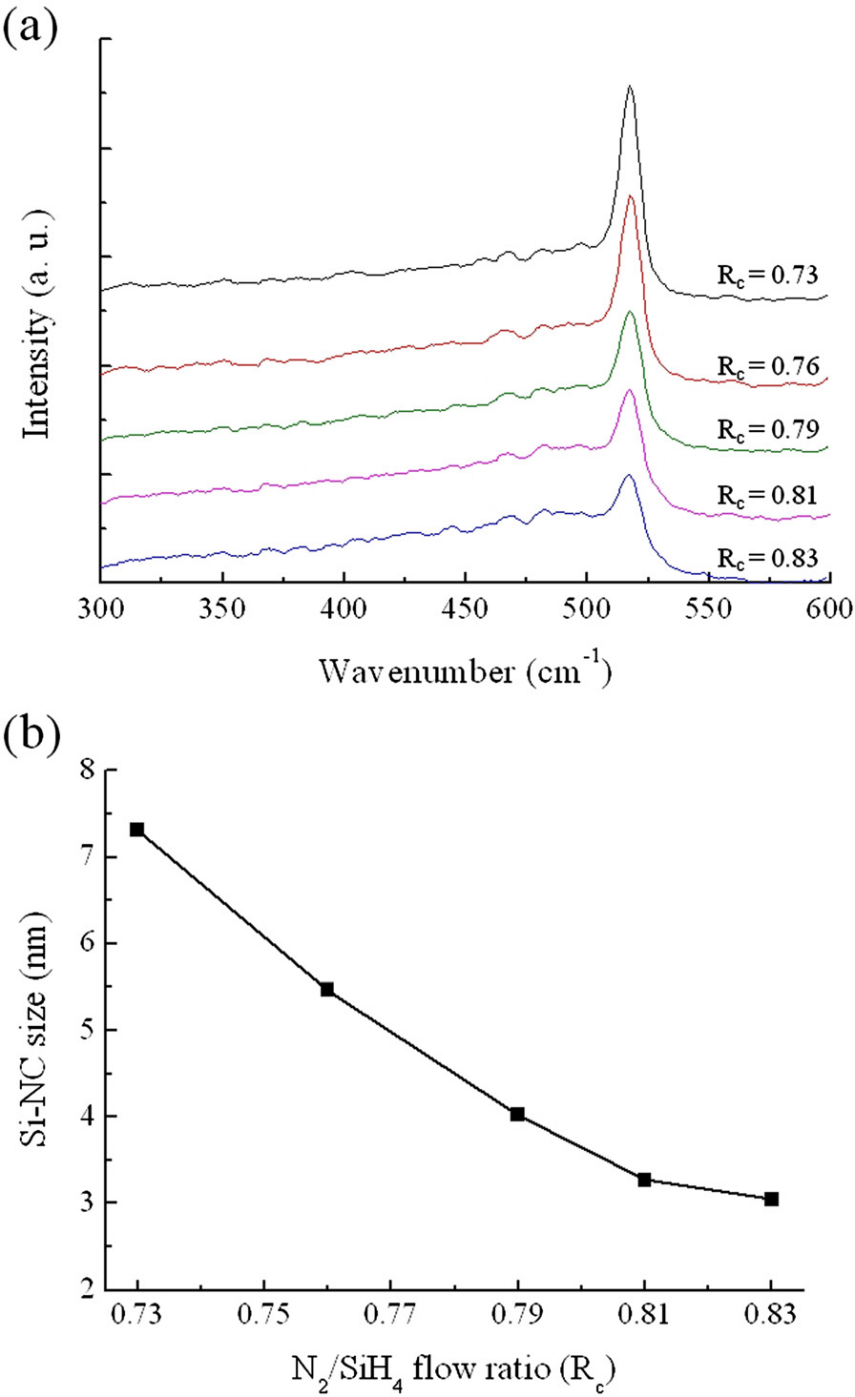
**Analysis of the crystallization behavior of P-doped Si-NCs/SiN**_***x***_**films. (a)** Raman spectra of P-doped Si-NCs/SiN_*x*_ films with various *R*_c_ values. **(b)** Average Si-NC size of the Si-NCs/SiN_*x*_ film as a function of the *R*_c_ value obtained by XRD data with the Scherrer equation.

**Figure 3:**
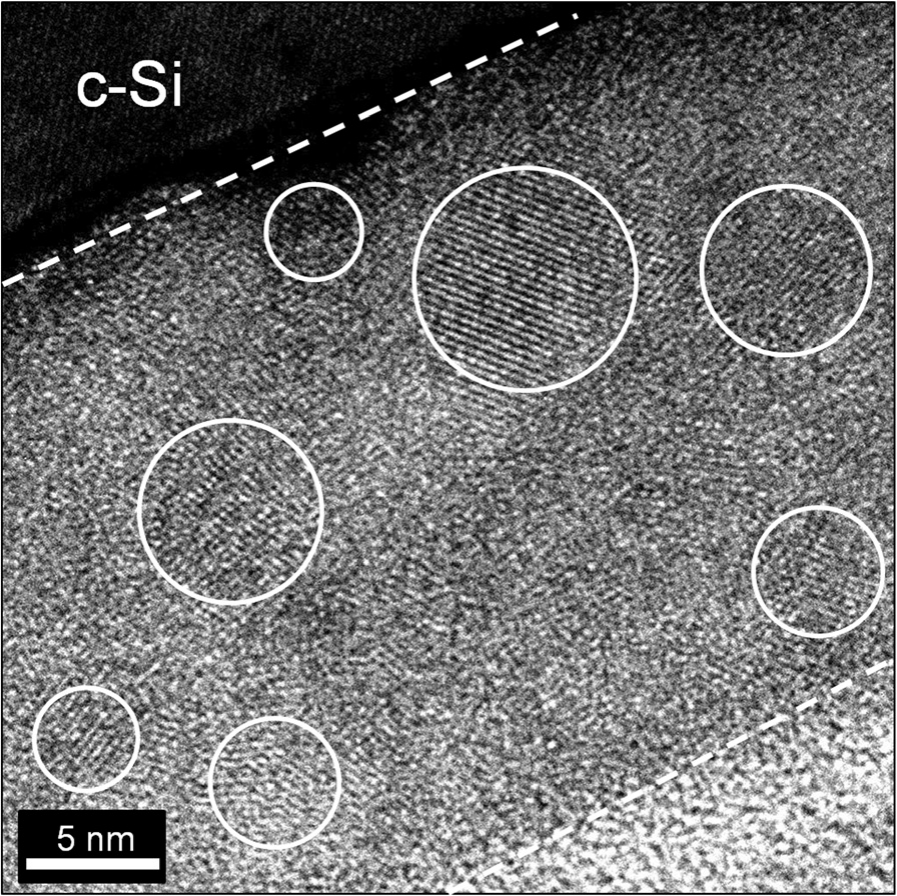
**Representative TEM image of the P-doped Si-NCs/SiN**_***x***_**film with *R***_**c **_**= 0.79.** The crystalline structure of Si-NCs is circled by white circles. Dashed lines indicate interfaces between the Si-NCs/SiN_*x*_ film and surrounding c-Si wafer and epoxy layer.

Figure 2a shows the Raman spectra of the P-doped SRN films with various *R*_c_ values after annealing at 950°C for 30 min. The peak corresponding to the c-Si mode (located between 510 and 520 cm^−1^) appears due to precipitation of Si-NCs in the films during annealing. As depicted in Figure 2a, the growing c-Si peak intensity with decreasing *R*_c_ value indicates that the volume fraction of Si-NCs increases with increasing excess Si concentration in the SRN films, which is consistent with XPS results shown in Figure 1c. In this study, the average Si-NC size was estimated from the XRD data with the Scherrer equation: *D* = *kλ* / *β*cos*θ*, where *D* is the average crystallite size, *λ* is the wavelength of the X-ray, *β* is the full width at half maximum (FWHM) of the diffraction peak, and *θ* is the Bragg angle [18]. The value of the correction constant *k* was usually taken equal to 0.9 for Si. Figure 2b shows the average Si-NC size of the Si-NCs/SiN_*x*_ film as a function of the *R*_c_ value. It is observed that the average crystallite size decreases from 7.3 to 3.0 nm for the Si-NCs/SiN_*x*_ films over the investigated range of N_2_/SiH_4_ flow ratio. High-resolution TEM was also used to confirm the formation of Si-NCs. Figure 3 shows a representative TEM image of the Si-NCs/SiN_*x*_ film with *R*_c_ = 0.79. The lattice fringes in the amorphous SiN_*x*_ matrix indicate the formation of Si-NCs. The size distribution of Si-NCs is in the range of 3 to 8 nm. The calculated average size of Si-NCs obtained from TEM images is consistent with that estimated from the XRD measurement.

**Figure 4:**
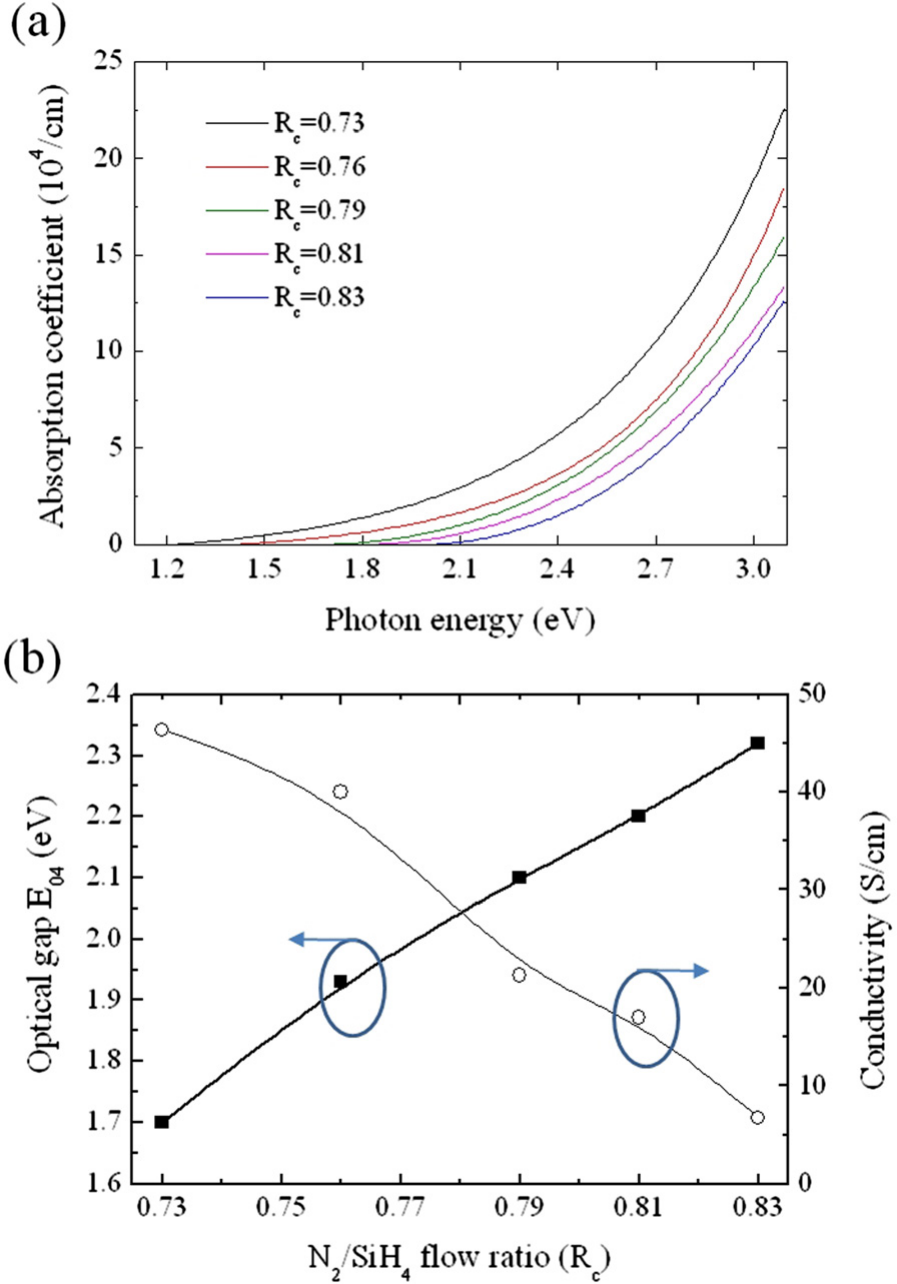
**Optical and electrical properties of P-doped Si-NCs/SiN**_***x***_**films. (a)** Absorption coefficients of the P-doped Si-NCs/SiN_*x*_ films versus the incident photon energy. **(b)** Optical gap E_04_ and electrical conductivity of P-doped Si-NCs/SiN_*x*_ films as a function of the *R*_c_ value.

In this work, the optical absorption of the P-doped Si-NCs/SiN_*x*_ film was evaluated using optical gap E_04_ defined as the energy at which the absorption coefficient is equal to 10^4^ cm^−1^. In order to obtain the energy E_04_, the extinction coefficient was deduced from ellipsometry measurements, and then the absorption coefficient *α* was calculated from the determined extinction coefficient *k* through the relation *α* = 4*πk* / *λ*, where *λ* is the wavelength. Figure 4a shows absorption coefficients of the P-doped Si-NCs/SiN_*x*_ films versus the incident photon energy. In addition, the electrical conductivity of the P-doped Si-NCs/SiN_*x*_ film was measured by the van der Pauw method at room temperature. The derived optical gap E_04_ and electrical conductivity are shown as a function of the N_2_/SiH_4_ flow ratio in Figure 4b. As the nitrogen content increases, the electrical conductivity decreases from 46.4 to 6.7 S/cm over the investigated range of N_2_/SiH_4_ ratio, while the opposite trend is observed for the optical gap E_04_, increasing with a gain of 0.52 eV. The Si-NCs/SiN_*x*_ film is considered as a two-phase heterogeneous material, consisting of low-resistivity Si-NCs needed for good carrier transport and the wide bandgap SiN_*x*_ matrix for high transparency. According to the effective medium approximation [19], the Si-NCs/SiN_*x*_ film can be schematized as an effective medium, and its physical properties (electrical conductivity and absorption coefficient) could be derived from the physical properties and volume fractions of each phase. Thus, the less conductive and more transparent material obtained with increasing nitrogen content could be ascribed to the reduction in volume fraction of Si-NCs, as depicted in Figure 2a. In addition, due to the quantum confinement effects [20], the shrinkage of the Si-NC size with increasing *R*_c_ value may result in bandgap expansion, which also leads to an increase in the effective optical gap of the Si-NCs/SiN_*x*_ film.

**Figure 5:**
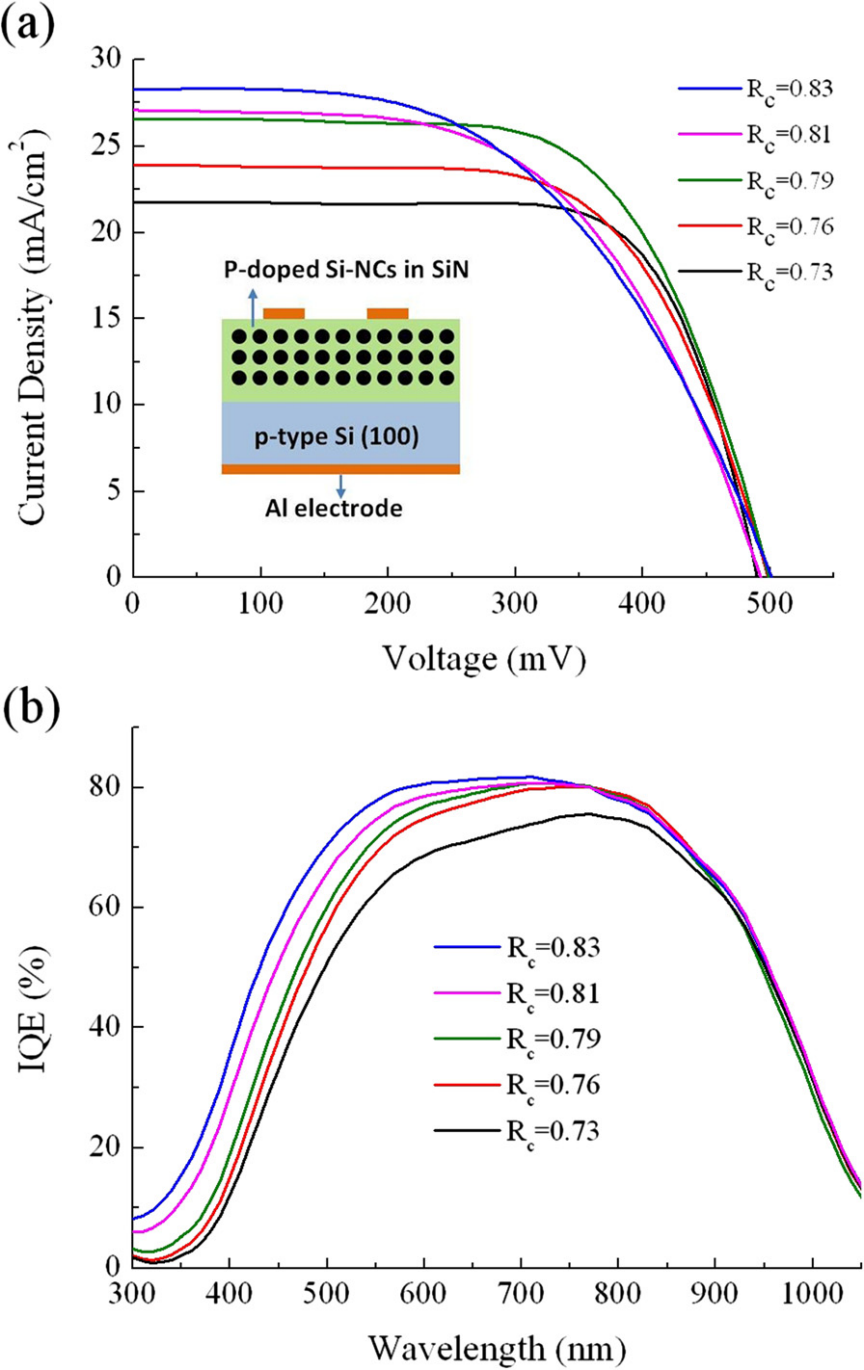
**Illuminated *J*-*V *characteristics and IQE of Si-NCs/sc-Si heterojunction solar cells. (a)***J*-*V* characteristics of Si-NCs/sc-Si heterojunction solar cells under air mass 1.5 illumination. The inset on the left bottom is a schematic of the fabricated Si-NCs/sc-Si heterojunction cell. **(b)** IQE of Si-NCs/sc-Si heterojunction solar cells with different *R*_c_ values.

**Figure 6:**
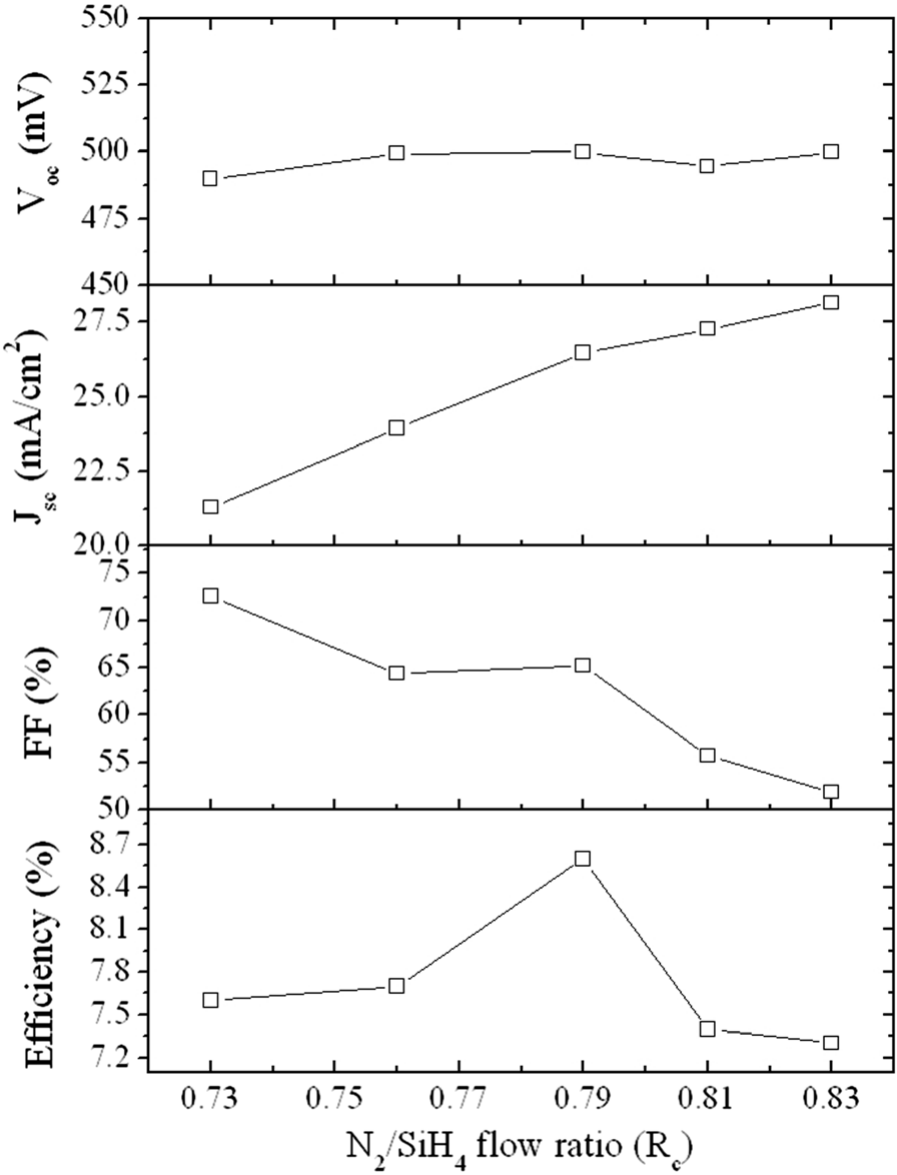
**One-sun illuminated cell parameters of Si-NCs/sc-Si heterojunction solar cells.** The *V*_oc_, *J*_sc_, FF, and efficiency of the fabricated Si-NCs/sc-Si heterojunction cells with different *R*_c_ values.

**Figure 7:**
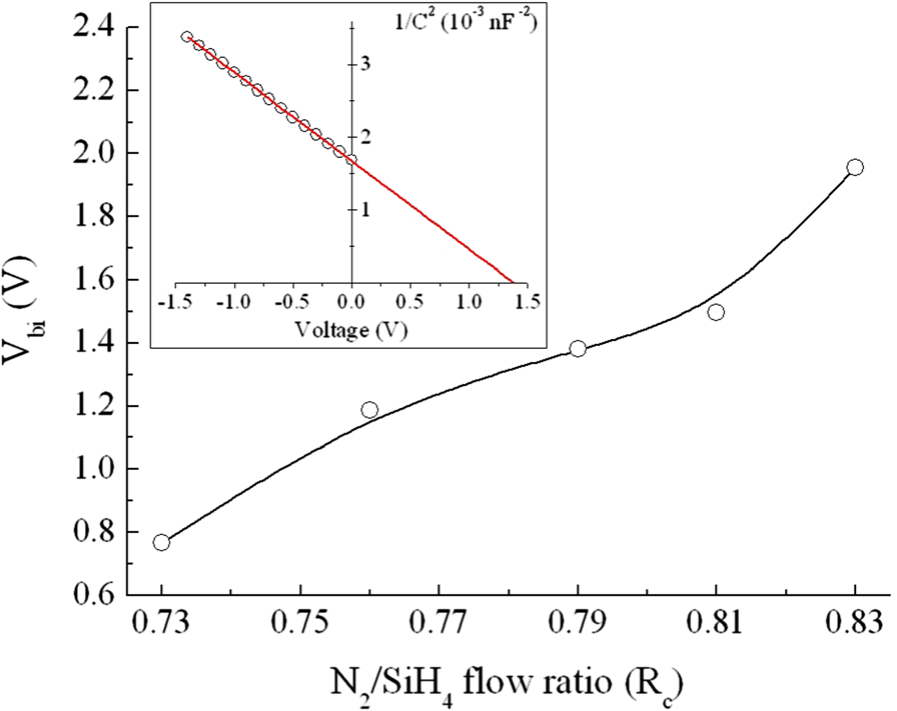
**Built-in potential of the Si-NCs/sc-Si heterojunction as a function of the *R***_**c **_**value.** The inset is an inverse capacitance-square plot of the *R*_c_ = 0.79 sample.

**Figure 8:**
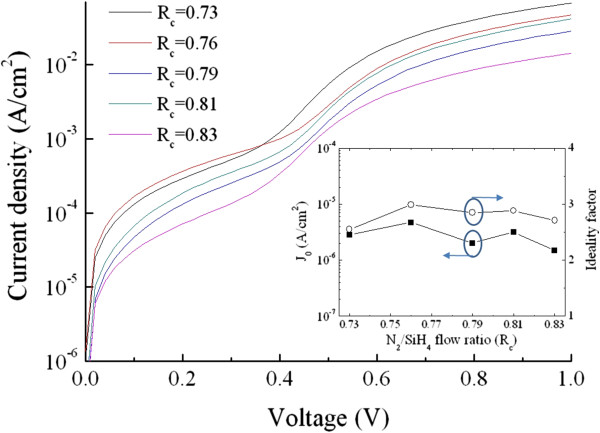
**Dark current density-voltage characteristics of Si-NCs/sc-Si heterojunction solar cells.** The inset shows the saturation current density *J*_0_ and ideality factor *n* as a function of the *R*_c_ value.

The P-doped Si-NCs/SiN_*x*_ layers with various *R*_c_ values were fabricated on top of p-type sc-Si substrates for fabrication of Si heterojunction solar cells, as shown in the inset of Figure 5a. This study concentrates on basic Si-NCs/sc-Si heterojunction solar cells without the designs or processes to enhance the conversion efficiency, such as surface texturing, anti-reflection coating and back-surface field. The illuminated *J*-*V* curves corresponding to each sample are displayed in Figure 5a, and their open-circuit voltage (*V*_oc_), short-circuit current density (*J*_sc_), fill factor (FF), and efficiency are shown in Figure 6 as a function of the N_2_/SiH_4_ flow ratio. The magnitude of *V*_oc_ is generally correlated to the built-in potential (*V*_bi_) of the junction, which could be influenced by the energy bandgap of the Si-NCs for the Si heterojunction solar cells. As shown in Figure 7, the *V*_bi_ of the P-doped Si-NCs/sc-Si heterojunction extracted from the capacitance-voltage characteristic increases from 0.77 to 1.95 V with increasing *R*_c_ value. This trend may be ascribed to the bandgap expansion of Si-NCs with the shrinkage of the Si-NC size, leading to an increase in *V*_bi_ at the junction, and thus, the Si heterojunction solar cell is expected to show a higher *V*_oc_ as *R*_c_ increases. However, in this study, the *V*_oc_ value is in the range of 0.49 to 0.5 for all Si heterojunction solar cells (shown in Figure 6), implying that *V*_oc_ is quite insensitive to the Si-NC size. Figure 8 shows dark *J*-*V* curves for the solar cells with different *R*_c_ values. Both the saturation current density (*J*_0_) and the ideality factor (*n*) were extracted by fitting the dark *J*-*V* curves at intermediate voltages (approximately 0.4 to 0.5 V) using a diode equation *J* = *J*_0_exp(*qV / nkT*), where *q* is the electron charge, *T* is the temperature, and *k* is the Boltzmann constant [21]. As shown in the inset of Figure 8, the values of *J*_0_ and *n* are in the ranges of 1.5 × 10^−6^ to 5 × 10^−6^ A/cm^2^ and 2.5 to 3 for all heterojunction solar cells, respectively. The large *n* value (*n* > 2), together with the high *J*_0_, indicates that the recombination current contributes significantly to the conduction process in the cells, which may be caused by trap-assisted tunneling or field-assisted recombination at point defects [22,23]. It has been reported that formation of charged defects would occur in SiN_*x*_ films after high-temperature annealing owing to the removal of hydrogen atoms [24,25]. Since the charged defect density in the annealed film should be proportional to the volume fraction of the SiN_*x*_ matrix, we suggest that the increase in the charge defect density would increase the probability of trap-assisted tunneling and thus compensate the enhanced *V*_bi_ effect with increasing *R*_c_ value, leading to similar *J*_0_, as well as *V*_oc_ for all heterojunction solar cells.

From Figure 6, the *J*_sc_ is increased from 21.3 to 28.2 mA/cm^2^ with increasing *R*_c_ value. This trend could be ascribed to the lower parasitic absorption in the Si-NCs/SiN_*x*_ film with a higher *R*_c_ value since the increasing Si-NC phase could result in a reduction in the optical gap of the film due to its higher absorption coefficient, as mentioned above (see Figure 4b). To better understand the difference in *J*_sc_ among the heterojunction solar cells with various *R*_c_ values, losses of the *J*_sc_ in the devices were investigated from their IQE data by spectral response measurements. As shown in Figure 5b, the heterojunction device with a higher *R*_c_ Si-NCs/SiN_*x*_ film shows a higher IQE in the short wavelength regime, especially for the wavelength range between 400 and 700 nm, while the IQE spectra in the infrared wavelength regime (>900 nm) are similar for all heterojunction solar cells, implying that recombination of photo-generated charge carriers in the absorber layer is almost the same in all heterojunction devices [26]. Moreover, as depicted in Figure 4a, the obvious variations in the absorption spectra of the P-doped Si-NCs/sc-Si films with various *R*_c_ values could be observed at photon energies above 1.8 eV (approximately <700 nm), which shows good correspondence with the trends in the IQE data. Therefore, it is speculated that the difference in *J*_sc_ losses among the devices could be attributed to the parasitic absorption in the emitter layer. More photons in the visible spectrum would be absorbed with increasing volume fraction of the Si-NCs in the P-doped Si-NCs/sc-Si film, leading to the limitation in the available solar spectrum in the device, as well as the degradation of the *J*_sc_.

**Figure 9:**
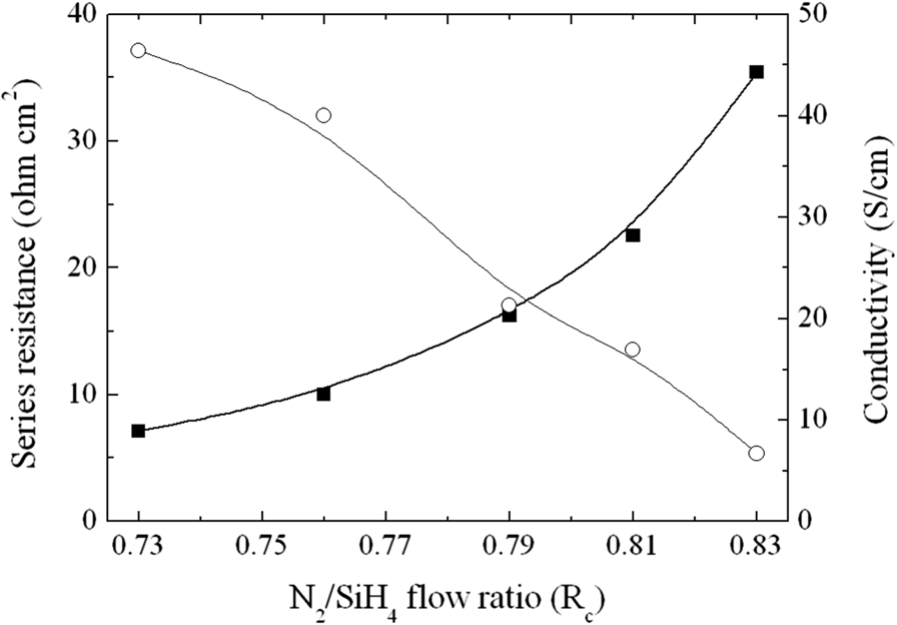
**Series resistance and electrical conductivity as a function of the *R***_**c **_**value.**

In contrast to the *J*_sc_, the FF decreases from 72.6% to 51.9% when increasing the *R*_c_ value, as depicted in Figure 6. The series resistance (*R*_s_) of the Si heterojunction solar cell was extracted from the dark *J*-*V* characteristic and shown in Figure 9 as a function of the *R*_c_ value. The fill factor of a solar cell depends upon the series resistance, saturation current density, and diode ideality factor. Here, the reduction in FF with increasing *R*_c_ value could be mainly attributed to an increase in *R*_s_ since the values of *J*_0_ and *n* are similar for all heterojunction solar cells, as shown in the inset of Figure 8. As depicted in Figure 9, the *R*_s_ of the Si heterojunction solar cell is highly correlated to the conductivity of the P-doped Si-NCs/sc-Si film. Thus, it could be speculated that the FF of the Si heterojunction solar cell strongly depends on the conductivity of the P-doped Si-NCs/SiN_*x*_ film. The maximum conversion efficiency is achieved from the device with N_2_/SiH_4_ ratio of 0.79 (shown in Figure 6), where the balance between *J*_sc_ and FF losses is optimized. The best heterojunction solar cell has 8.6% conversion efficiency, with a *V*_oc_ of 500 mV, *J*_sc_ of 26.5 mA/cm^2^, and 65.2% in fill factor. While the data obtained is based on our preliminary fabrication of Si-NCs/sc-Si heterojunction cells, further improvement in fabrication of Si-NC emitters (layer thickness, deposition and doping conditions, etc.) and related process parameters is likely to improve the photovoltaic efficiency.

## Conclusions

In this report, we have investigated the feasibility of using P-doped Si-NCs/SiN_*x*_ films as emitters on p-type sc-Si substrates for fabrication of Si-based heterojunction solar cells. From XPS analysis of the P-doped Si-NCs/SiN_*x*_ films, the P 2*p* signal only attributed to Si-P or P-P bonds indicates that the P atoms may exist inside Si-NCs. The electrical and optical properties of the P-doped Si-NCs/SiN_*x*_ material are strongly influenced by its chemical composition (N/Si ratio). The optical gap E_04_ is enhanced with increasing nitrogen content, while the conductivity is deteriorated. These trends could be interpreted by a bi-phase model, where the SiN_*x*_ phase contributes to the optical gap enhancement and the Si-NC phase promotes charge carrier transport. Therefore, the *J*_sc_ is increased with increasing N/Si ratio in the Si-NCs/SiN_*x*_ layer, while the FF is reduced. The best cell parameters obtained are *V*_oc_ of 500 mV, *J*_sc_ of 28.2 mA/cm^2^, FF of 65.2%, and conversion efficiency of 8.6% from the heterojunction solar cell with a *R*_c_ = 0.79 Si-NCs/SiN_*x*_ emitter. Further device optimization is required to improve the photovoltaic efficiency.

## Competing interests

The authors declare that they have no competing interests.

## Authors’ contributions

PJW carried out the material and device preparation and drafted the manuscript. YCW carried out the material and device characterization. ICC conceived of the study and participated in its design and coordination. All authors read and approved the final manuscript.
